# A systematic proximity map of the centriole-cilia interface

**DOI:** 10.1186/2046-2530-4-S1-P72

**Published:** 2015-07-13

**Authors:** J Gonçalves, B Mojarad, G Gupta, E Coyaud, B Raught, L Pelletier

**Affiliations:** 1 LAB, Lunenfeld-Tanenbaum Research Institute, Mount Sinai Hospital, Toronto, Ontario, Canada

## Objective

Eukaryotic cilia/flagella are dynamic microtubule (MT)-based organelles. The ciliary axoneme is built from a radial scaffold of 9 MT doublets, plus a central MT pair, in the case of motile cilia. The axoneme is templated by a basal body (BB)/centriole derived from the mother centriole, in the case of animal cells, which presents sub-distal and distal appendages critical for cilia formation. In vertebrates, different cilia types fulfil diverse functions, critical for embryonic development and homeostasis of adult tissues. Cilia malfunction causes ciliopathies, but despite their biomedical implications, their molecular composition and mechanisms underlying their biogenesis still remain poorly defined.

## Methods

In order to dissect the mechanisms involved in primary cilia assembly and disassembly we decided to focus on the mother centriole appendages and the TZ and define the interaction networks of their known components (~60 proteins) using a new mass spectrometry approach called BioID based on the proximity-dependent biotinylation of proteins by a promiscuous biotin ligase fused to the protein of interest.

## Results

With this approach we have identified near neighbors and potential new interactors of ~60 known components of the centriole-cilia interface and the ciliary transition zone which have been tested for their involvement in centrosome and cilia biology using automated high-throughput/high-resolution screens and precise morphometric measurements.

## Conclusion

We identified several new regulators of ciliogenesis a subset of which we have characterized. The potential role of the most promising candidates in the potential onset of clinical features of ciliopathies will also be discussed.

